# Book Review: Language Education and Emotions: Research Into Emotions and Language Learners, Language Teachers and Educational Processes

**DOI:** 10.3389/fpsyg.2021.735403

**Published:** 2021-07-28

**Authors:** Hua Wang

**Affiliations:** School of Journalism, Nanjing University of Finance and Economics, Nanjing, China

**Keywords:** language education, emotions, language learner, language teacher, educational processes

Research on the interplay between language education and emotions has piqued much interest among language practitioners and researchers, culminating in tremendous growth. It has been corroborated that while positive emotions facilitate language education, negative emotions debilitate the process of language learning. The present compendium, *Language Education and Emotions: Research into Emotions and Language Learners, Language Teachers and Educational Processes*, edited by Mathea Simons and Tom F.H. Smits, foregrounds the role of both positive and negative emotions by integrating the three perspectives of language learners, language teachers, and educational processes through the reconciliation of robust theoretical underpinnings and rigorous evidence-based studies.

This compendium includes a Preface, an Introduction, three parts, encompassing 10 chapters, and a Conclusion. In the Preface, the editors argue that emotions in language education can be viewed from three perspectives, including learners, teachers, and educational processes. In the Introduction, the author cogently argues that “one of the challenges in teaching contexts today is to provide more ways to educate all aspects of the student, including greater attention to the affective aspects as well as the cognitive” (p. 14). Consequently, this chapter conceptualizes the fundamental concepts and justifies why affective factors can have a pervasive impact on successful language education.

Part I, entitled *Emotions and the Language Learner*, includes four chapters. Chapter 2 appraises the prominence of emotion-regulation strategies (ERSs) concerning the students' learning outcomes as well as emotional and cognitive growth and enumerate different ERSs with their frequencies. Drawing on the mixed-methods research, Chapter 3 scrutinizes how 19 Chinese-as-a-foreign-language (CFL) learners' self-regulated learning strategies can affect their self-efficacy in a flipped classroom. The author concludes that “This individualized approach to improving learners' SRL strategies was successful in helping students find and apply effective strategies to maximize their learning” (p. 49).

Looking at foreign language anxiety (FLA), Chapter 4 aims to shed light on the nature of Spanish learners' FLA in an interactive online environment. The authors reported that the main sources of FLA are the speaking partner and the activities of the course. Besides, they found that “more anxiety-reducing sources of FLA were identified as opposed to anxiety-triggering ones” (p. 71), substantiating the idiosyncrasy and dynamicity of FLA. Chapter 5 unpacks how professional and personal recognition, appreciation, and the willingness to adopt communication patterns in the host country can influence the sensibility of Spanish migrant language learners to recognize forms of interaction of the guest culture. The author concludes that “it would seem advisable and constructive to include raising awareness of culture-specific traits in language teaching” (p. 91).

Part II, titled *Emotions and the Language Teacher*, includes three chapters that probe into the interconnectedness of emotions and the language teacher. Utilizing self-confrontation interviews, Chapter 6 scrutinizes the effects of emotions on the development of novice EFL teachers' professional identity. The author highlights that the use of logbooks can make novice teachers reflective practitioners and assist them to “draw positivity out of negative-valence emotions” (p. 107). Drawing on both quantitative and qualitative data collection instruments, Chapter 7 aims to unpack the extent to which non-native foreign language teachers experience anxiety, find out the role of the target language, and explore the potential antecedents of feelings of foreign language teaching anxiety on 38 pre-service and early-career in-service teachers. The results illuminate that teachers like advanced learners experience anxiety and besides those sources of anxiety that have been acknowledged in the literature, the participants' cultural background can be considered as a source of FLA, and provide some coping mechanisms. Chapter 8 scrutinizes the underlying influences of a teacher's self-disclosure (SD) on nine Iranian students' foreign language enjoyment through an ethnographic case study. Findings indicated that “the learners interpreted the teacher's SD as an attempt to be open about herself, to make a personal connection and to be supportive. They also reported that some dimensions such as being positive, human, honest and social were associated with the teacher's SD” (p. 140).

Part III, *Emotions and the Educational Process*, contains three chapters. Chapter 9 focuses on the role of verbalizing emotions by investigating how social distance and dominance impact the lexical choices among Austrian classroom learners of English and British English L1 users make to talk about emotions in English. The chapter reports that cultural and linguistic proximity are utilized by foreign language learners when they verbalize their emotions, taking into consideration that the frequency of emotion-naming assertions and the richness of the emotion lexicon are similar. Chapter 10 explores the impact of two pronunciation learning strategies, namely record and listen vs. simply repeating without recording, on 12 ESL undergraduate and graduate students' level of motivation, sense of progress, and self-confidence. The results revealed that both strategies were effective, the record and listen strategy were more effective on learners' positive emotions. The penultimate chapter examines emotions in content and language integrated learning (CLIL) at a German high school. The chapter concludes that code-switching in CLIL can lead to both emotional and rational learning. In the closing chapter, the author recapitulates the status qua of emotions and language education, integrates the three perspectives of learners, teachers, and the educational process by highlighting “how the sources of emotions in learners also play a role in the emotions of teachers and how the emotions of both learners and teachers interact in dynamic ways” (p. 218), and discusses the takeaways for theory advancement and future studies.

As an applied linguist and a practitioner, I feel that this volume is meritorious for language educators, students, and researchers in that it brings together three perspectives, namely learners, teachers, and educational processes into account. Besides, the chapters provide theoretically illuminating and empirically convincing studies that not only confirm the previous studies but also pave the way for future research. Had the editors included one chapter on boredom in EFL/ESL classes, it would have been insightful.

## Author Contributions

The author confirms being the sole contributor of this work and has approved it for publication.

## Conflict of Interest

The author declares that the research was conducted in the absence of any commercial or financial relationships that could be construed as a potential conflict of interest.

## Publisher's Note

All claims expressed in this article are solely those of the authors and do not necessarily represent those of their affiliated organizations, or those of the publisher, the editors and the reviewers. Any product that may be evaluated in this article, or claim that may be made by its manufacturer, is not guaranteed or endorsed by the publisher.

